# The evolution of pertactin expression in Belgian circulating *B. pertussis* strains, pre- and post-COVID-19

**DOI:** 10.1128/spectrum.01535-25

**Published:** 2026-03-30

**Authors:** Helena Martini, Oriane Soetens, Vili Niinikoski, Alex-Mikael Barkoff, Qiushui He, Denis Piérard, Eveline Van Honacker

**Affiliations:** 1 Department of Microbiology, National Reference Center for *Bordetella pertussis*, Universitair Ziekenhuis Brussel 60201, Brussels, Belgium; 2 Vitality Research Group, Vrije Universiteit Brussel (VUB) 70493, Brussels, Belgium; 3 Faculty of Medicine and Pharmacy, Vrije Universiteit Brussel (VUB) 74878 , Brussels, Belgium; 4 European Reference laboratory for Public Health on Diphtheria and Pertussis (EURL-PH-DIPE), Turku, Finland; 5 Research Center for Infections and Immunity, Institute of Biomedicine, University of Turku 169300, Turku, Finland; 6 InFLAMES Research Flagship Center, University of Turku 8058, Turku, Finland; National Chung Hsing University, Taichung, Taiwan

**Keywords:** whooping cough, pertussis, pertactin, *Bordetella pertussis*

## Abstract

Prior to the COVID-19 pandemic, *Bordetella pertussis* strains not producing pertactin were globally increasing, especially in countries using the acellular pertussis vaccine. Subsequently, during the pandemic, global pertussis incidence dropped to extreme lows, likely due to non-pharmaceutical interventions (e.g., social distancing and face masks). During 2022–2024, many countries—including Belgium—saw a re-emergence of the disease. The newly circulating strains are mostly pertactin-expressing. Pertactin (Prn) antigen-expression testing was performed on 432 Belgian isolates collected in 2014–2023. Whole-genome sequencing for virulence typing and phylogenetic analysis was performed on 416 isolates. Before COVID-19, Prn-negative strains in Belgium went from making up less than 20% of circulating strains to being in the majority from 2017 to 2020. In 2022 and 2023, during the post-COVID re-emergence, all tested strains expressed pertactin. Further typing showed a variety of different mutations of the *prn* gene and/or its promoter region in the pre-pandemic Prn-negative strains. In phylogenetic analysis, these do not cluster together. Prn-positive *B. pertussis* strains have replaced Prn-negative ones in Belgium since the post-COVID-19 re-emergence. This could potentially be an effect of reduced population immunity, allowing some strains to spread rapidly with less selective pressure for pertactin deficiency. Further research and surveillance should be done to identify or refute other potential factors influencing pertactin expression.

**IMPORTANCE:**

During the COVID-19 pandemic, non-pharmaceutical intervention measures prevented the spread of other diseases, such as whooping cough (caused by *Bordetella pertussis*). Now that the use of anti-COVID measures has reduced, many countries have seen a return of infections with *B. pertussis*. Whereas pre-pandemic, a large part of these strains did not express pertactin, a component used in the pertussis vaccine, now most strains do. A large part of Belgian *B. pertussis* strains did not express pertactin before the COVID-19 pandemic. We found several different genetic mechanisms as the cause of this. During the pandemic, cases dropped to almost non-existent. Upon their return around 2023, strains showed less genetic diversity, and all expressed pertactin. Our findings illustrate how pertussis epidemiology can be unpredictable and change quickly. Genetic changes in the bacteria can have an effect on vaccine effectiveness and immunity; therefore, it is important to study these changes and maintain epidemiological surveillance.

## INTRODUCTION

As has been the case for many respiratory diseases, the incidence and epidemiological characteristics of pertussis have been greatly affected by the COVID-19 pandemic. Prior to its start in 2019–2020, *Bordetella pertussis* incidence all over the world generally showed a cyclical pattern, with peaks and lows every 3–5 years (1). On the level of virulence characteristics, one of the most important observations of the last couple of years prior to 2020 was the global increase of *B. pertussis* strains not producing the vaccine antigen pertactin, especially in countries using the acellular pertussis (aP) vaccine (2–4).

The Belgian vaccination schedule includes primary vaccination at 2, 3, and 4 months of age, followed by booster vaccination at 15 months, 5–6 years, and 14–16 years (as well as adult boosters every 10 years). The aP vaccine has been in use in Belgium since 1999, although the whole-cell pertussis (wP) vaccine was still used for the three primary doses (2). A non-pertactin-containing aP vaccine replaced the wP vaccine fully in 2001. From 2004 until 2014–2015, the primary vaccination provided freely by the Belgian health services contained pertactin; in 2014 (Flanders) or 2015 (Wallonia and the Brussels Capital Region), a switch was made once more to non-pertactin-containing vaccines (5). In 2023, Wallonia and the Brussels Capital Region once more switched to a pertactin-containing alternative for primary vaccination (6). A detailed overview of the vaccines offered in Belgium can be found in Table S1 at https://doi.org/10.6084/m9.figshare.31557271. Vaccination coverage in Belgium reaches ≥97% for completion of the first three doses and ≥92% for completion of the first four doses (numbers for all three regions, 2012–2021) (7).

During the COVID-19 pandemic, global pertussis incidence dropped to extreme lows. The main causing factor of this drop was very likely the prominence of non-pharmaceutical interventions against the spread of respiratory infection, such as social distancing and the wearing of face masks. A reduction in non-COVID medical testing, leading to less pertussis diagnoses, may perhaps also have played a role (8, 9).

**Fig 1:**
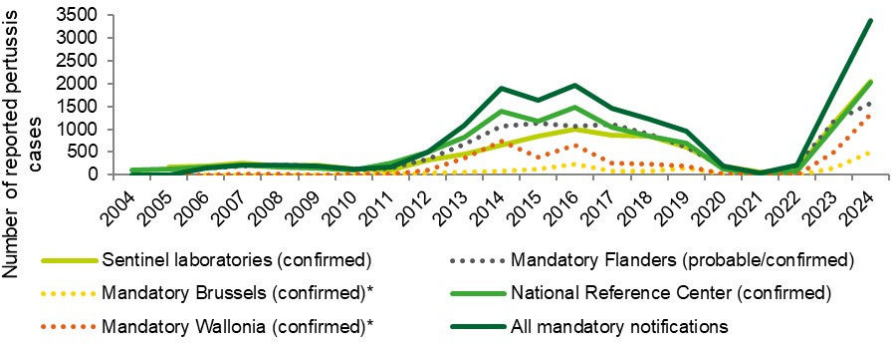
Cases of pertussis reported in Belgium, by source. *Note that the rules for mandatory notification differ between the regions. Flanders: all possible, probable, and confirmed cases. Brussels 2004–2019: all possible, probable, and confirmed cases. Brussels 2020–2024: all confirmed cases. Wallonia 2004–2019: all possible, probable, and confirmed cases. Wallonia 2020–2023: only confirmed cases in patients under the age of 3 years. Wallonia 2024: all confirmed cases. Source: Peeters I, Lizroth A, Desombere I, Martini H, Piérard D, Boulouffe C, et al. Epidemisch kinkhoest rapport: januari tot en met juni 2024. Belgium: Sciensano; 2024. Available from: https://www.sciensano.be/nl/biblio/epidemisch-kinkhoest-rapport-januari-tot-en-met-juni-2024 (13*).*

During the years 2022–2024, many countries saw a re-emergence of the disease. In some countries, this has, by now, stabilized to pre-COVID levels; in others, the re-emergence is still ongoing (10–12). Belgium saw its peak mostly in the second half of 2023 and the first half of 2024. Figure 1 shows the cases in Belgium as reported by different sources: the sentinel laboratories, the mandatory notifications, and the National Reference Center for *Bordetella pertussis* (from here on referred to as the NRC or the Belgian NRC) (13).

The Belgian NRC provides laboratory confirmation of pertussis cases from the whole country, through real-time PCR (qPCR) or serology. The NRC also attempts to culture as many strains as possible from PCR-positive samples in order to enable further typing and characterization. Through virulence typing, the observation was made that strains collected before COVID-19 had been increasingly lacking pertactin expression, but those collected during the pertussis re-emergence were all pertactin positive.

This study aims to describe the evolution of pertactin expression in Belgian circulating *B. pertussis* strains during 2014–2023 and assess the changes involved on the genetic level.

## MATERIALS AND METHODS

### Laboratory diagnosis and strain collection

Culture was attempted on all clinical samples found positive for *B. pertussis* by PCR at the NRC, barring some exceptions (transport media unsuitable; high Ct values). Clinical samples were cultured on laboratory-prepared Regan-Lowe agar plates and incubated for 11 days at 35°C (±2°C) under increased humidity. Suspect colonies were identified by matrix-assisted laser desorption/ionization time-of-flight mass spectrometry, and—if positive for *B. pertussis*—re-isolated into pure growth. The pure clinical strains were added to the UZ Brussel (UZB) collection and stored at −80°C.

### Pertactin expression testing

Expression of pertactin was determined using a monoclonal antibody-based enzyme-linked immunosorbent assay (ELISA) (14). The strains isolated from samples collected from 2014 to 2021 were tested at the UZB, and the strains from samples collected in 2022 and 2023 were tested at the University of Turku in Finland, following the same protocol. The total number of isolated strains included was 432.

### Whole-genome sequencing

Whole-genome sequencing (WGS) was performed by the Brussels Interuniversity Genomics High Throughput core (www.brightcore.be), using Illumina technology. The Nextera XT DNA Library Prep Kit was used for library preparation. Sequencing depths between 26 and 3,582 were obtained. Raw reads were assembled using SPAdes, with automatic k-mer selection based on read length and with repeat resolution enabled. The resulting assemblies were cleaned up by filtering out contigs shorter than 500 base pairs or with a coverage lower than 5. The quality of the fasta files was then assessed using Quast (v5.0.2); minimum quality criteria for WGS data of *B. pertussis*, as outlined by Institut Pasteur’s BIGSdb, were upheld (https://bigsdb.pasteur.fr/bordetella/genomes-quality-criteria/) (15).

### Virulence typing

BIGSdb’s Sequence Tag Scanner tool was used for the typing of the pertactin gene (*prn*). When an exact match for a known *prn*-allele was not found, BLAST against the *prn2* reference sequence was used to investigate further. In partial hits on different contigs, flanking sequences were investigated, allowing the resolution of insertions and partial deletions. BLAST was also used to search for the pertactin promoter (pr-PRN-Bp) and a known mutation where part of this promoter is inversed (pr-inv-PRN-Bp) (16).

### Phylogenetic analysis

The phylogenetic relationship between the sequenced strains (*n* = 416) was analyzed by generating a cgMLST-based minimum spanning tree using the BIGSdb GrapeTree plugin and the MSTreeV2 method (17). A logarithmic scale was used, and branch lengths over 50 were shortened for visualization purposes.

## RESULTS

### Epidemiology and strain collection

Over the 10-year period from 2014 until 2023, the NRC performed 25,573 qPCRs and 34,739 serological assays for the laboratory diagnosis of *B. pertussis*, confirming 2,980 and 5,057 cases, respectively. Accounting for overlap (cases confirmed by both methods), 7,900 cases were confirmed by the NRC over the whole decade. Detailed numbers per year are shown in Table S2 at https://doi.org/10.6084/m9.figshare.31557271. For 2,834 of the 2,980 PCR-positive cases, culture was performed, resulting in the isolation of 747 unique strains (26%). Out of 2,980 cases, 1,774 originated in Flanders (of which 21% positive in culture), 712 cases originated in Wallonia (24% positive in culture), and 477 originated in the Brussels-Capital Region (35% positive in culture).

### Pertactin expression testing

**Fig 2:**
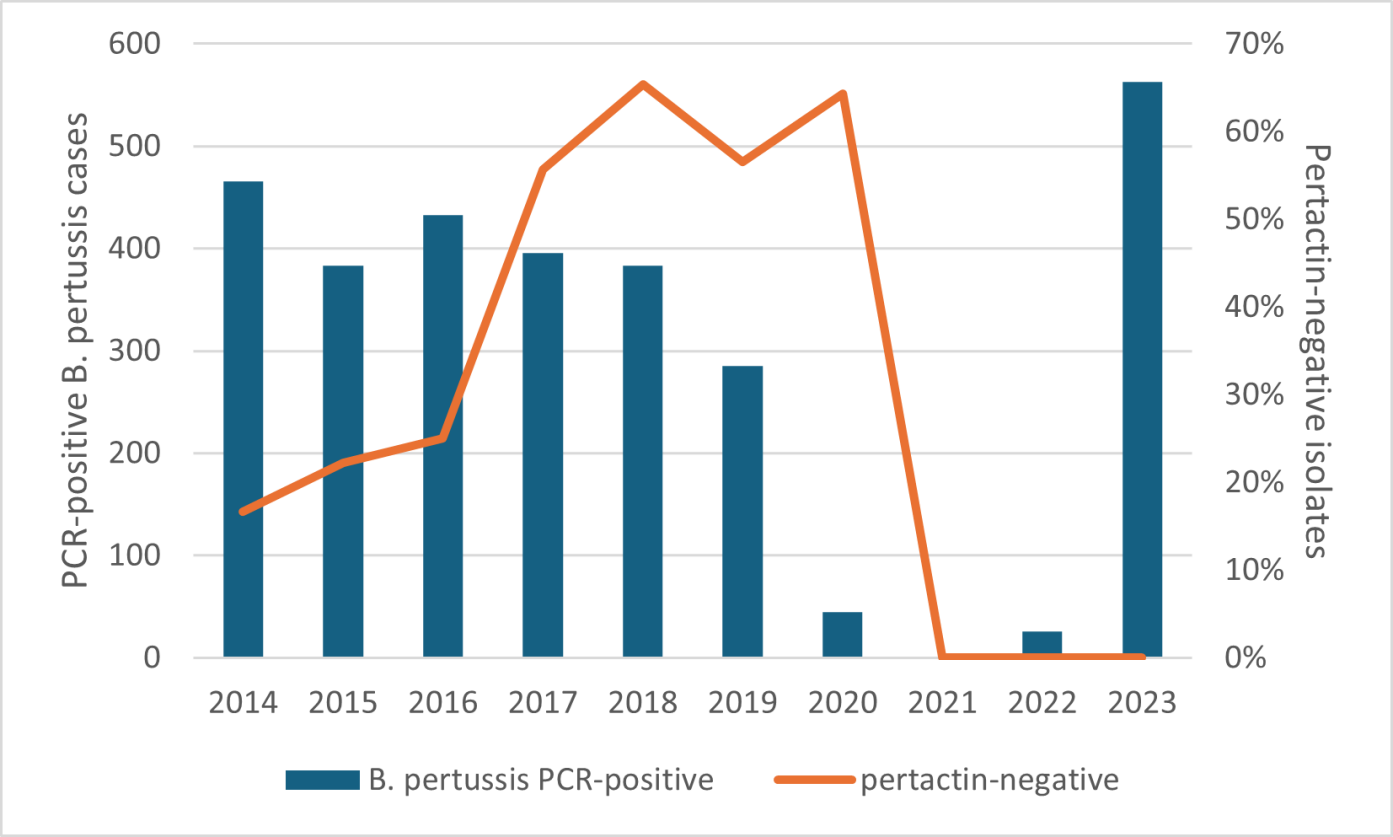
The orange line represents the percentage of ELISA-tested strains negative for pertactin expression (scale to the right). In order to place this evolution in the context of pertussis epidemiology, the blue columns were added, representing the total amount of PCR-positive cases of *B. pertussis* (scale to the left) confirmed by the NRC.

Before 2017, pertactin expression testing was only performed on a limited selection of strains (*n* = 50/359). At 20% of the tested strains, pertactin-negative strains made up the minority. During the period 2017–2020 (tested strains *n* = 288/290), they made up a larger part of the total, peaking at 65% in 2018. No strains were collected in 2021. In 2022 and 2023, all tested strains (*n* = 94/98) expressed pertactin. Figure 2 shows the evolution of pertactin expression over the years.

### Virulence typing

Out of the 432 strains for which pertactin expression was determined, 414 underwent WGS for further typing. Out of these, the pertactin-positive strains (*n* = 234) were, of course, all in possession of the *prn* gene. The *prn2* allele made up the vast majority of these (*n* = 213 or 91%). The other alleles that were found are *prn1*, *prn4*, *prn9*, *prn15*, and a new allele *prn160*.

The pertactin-negative strains (*n* = 180) showed some variation. Different mechanisms were found to be the cause of the lack of pertactin expression (detailed descriptions in Table S3 at https://doi.org/10.6084/m9.figshare.31557271):

- **An insertion of IS*481* in the *prn* gene**. This mutation occurred the most frequently, in 67 out of 180 strains.
- **A large deletion of part of the *prn* gene and *prn* promoter region**. Two different types were found, one deletion of 1,632 base pairs (bp)—found in 45 strains, the other of 1,083 bp—found in one strain.
- **Point mutations or single-nucleotide polymorphisms (SNPs), leading to a premature stop codon**. Three different allele types presenting such an SNP were found in 16 different strains.
- **A partial inversion of the *prn* promoter**. This 22 kb inversion was found in 36 strains, which all had an intact *prn2* gene.
- **A deletion in the *prn* promoter region**. Three strains showed an intact *prn2* gene but a partial deletion of the *prn* promoter.

Finally, in 12 out of 180 pertactin-negative strains, no genetic mechanism was found explaining the lack of pertactin expression. These 12 strains were in possession of an intact *prn2* gene and *prn* promoter.

**Fig 3:**
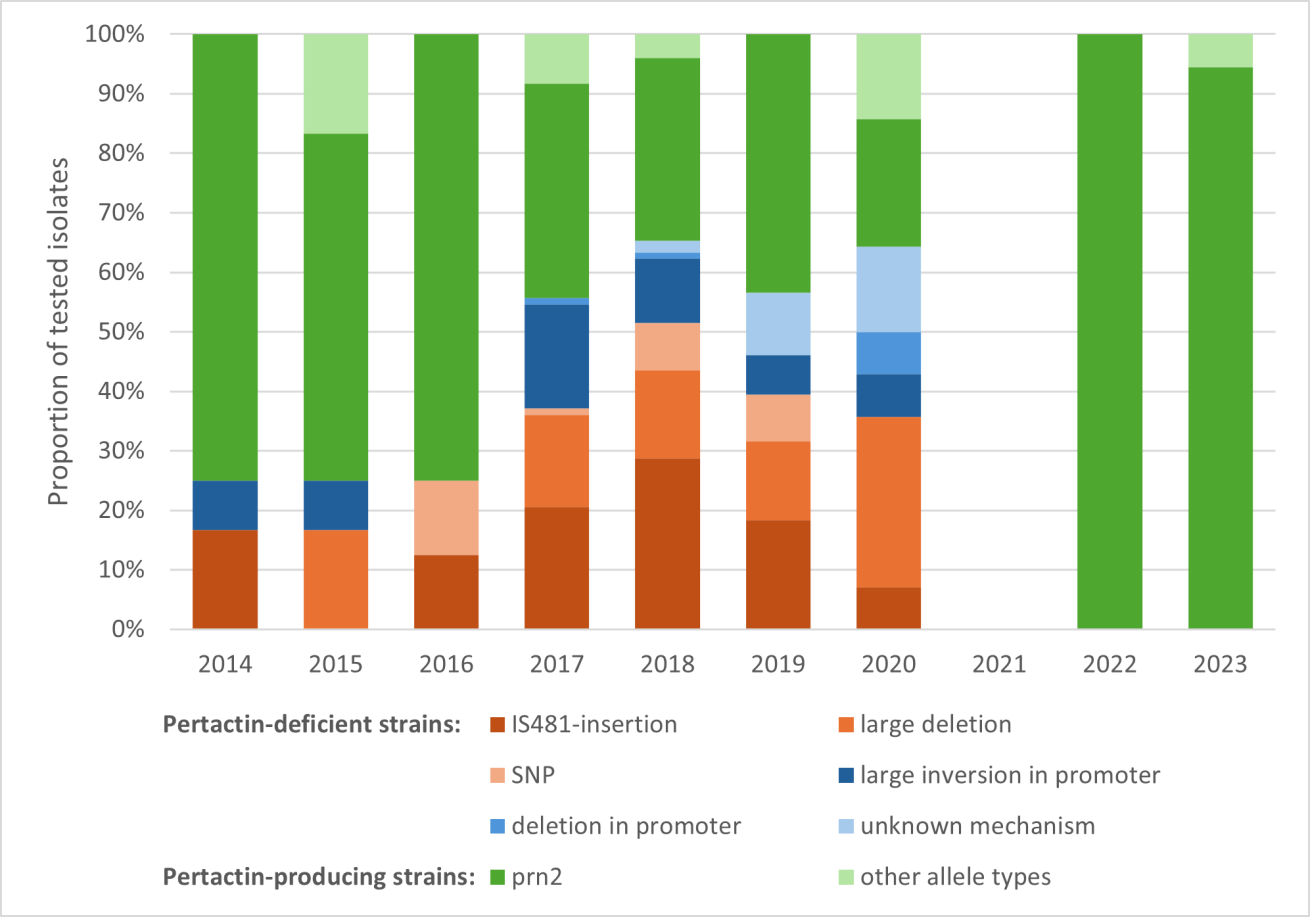
Distribution of the different *prn* types and mutations found each year. The green colors represent pertactin-positive strains, dark green *prn2* specifically, and light green all other allele types. The red colors represent pertactin-negative strains with a mutated *prn* gene. The blue colors represent pertactin-negative strains with an intact *prn* gene (all *prn2*).

Figure 3 graphically represents the distribution of the different *prn* types and mutations per year.

### Phylogenetic analysis

**Fig 4:**
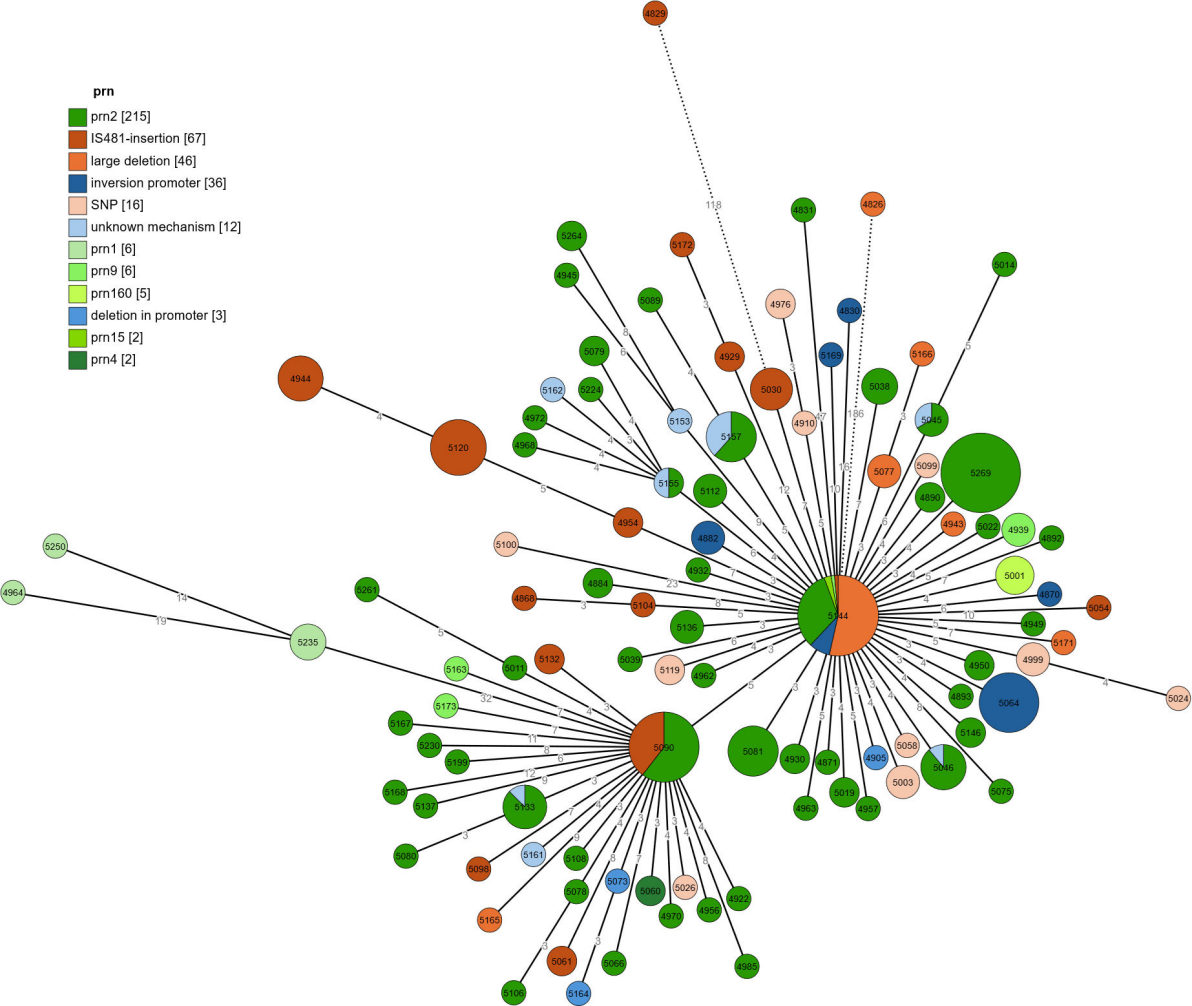
Minimum spanning tree based on cgMLST of the sequenced strains. Branch lengths are in logarithmic scale and represent the number of allelic mismatches. Branches longer than 50 are shortened (dotted lines). Nodes with two or fewer allelic mismatches are collapsed. Node labels are of the BIGSdb IDs. The colors represent the different *prn* types for Prn-positive strains and the different mechanisms for non-expression for Prn-negative strains (similar colors were used as in Fig. 3).

Figure 4 shows a cgMLST-based phylogeny of the sequenced strains. The different *prn* and promoter types and mutations do not cluster together but are spread out over the phylogenetic tree. We also performed phylogenetic analysis based on regions and age; however, no clustering was observed.

## DISCUSSION

The COVID-19 pandemic—and the non-pharmaceutical interventions it brought on—has had an overwhelming effect on pertussis incidence worldwide. A steep decrease in the number of reported infections was seen in countries all over the world, including Belgium (8, 9). During this period, a resurgence of the disease after the lifting of distancing and masking measures had been predicted. The expected re-emergence started to occur in many countries in 2023–2024 and came on fast and strong, with numbers often exceeding those from the pre-COVID period. A likely explanation for this is a reduction in population immunity after such a long period of little to no circulation of *B. pertussis* (10–12).

Upon the return of circulating *B. pertussis* strains, routine virulence gene typing led to a remarkable observation: whereas many countries had been noting a steady increase in strains not expressing pertactin during the pre-pandemic period, the post-pandemic strains tended to be almost exclusively pertactin producing (2, 10, 11).

In Belgium, *B. pertussis* isolates collected by the NRC had been the majority pertactin-negative since 2017, until the post-COVID-19 period, when pertactin-negative strains disappeared completely. All 94 isolates collected in 2022 and 2023 were found to produce pertactin and be in possession of the common *prn2* allele type in most cases (95%).

Pertactin is a highly immunogenic virulence factor and promotes adhesion to tracheal epithelial cells. Anti-Prn antibodies are associated with protection against pertussis (18); they were found to be crucial for *B. pertussis* phagocytosis (19) and are important bactericidal antibodies (20). In addition, pertactin was found to induce type-specific antibodies when sera of patients infected by strains with different *prn* alleles (*prn*1, *prn*2, and p*rn*3) were tested (21).

A closer inspection of the genotypes shows that the pre-pandemic pertactin-negative strains carried several causing mechanisms for pertactin deficiency, suggesting that their current absence is not due to the disappearance of one particular sublineage. A possible explanation that has been offered is the switch in several countries from acellular vaccines that contain pertactin to ones that do not. In Belgium, this switch was already made around 2014–2015, while the number of pertactin-negative strains continued to rise steeply for a few more years afterward. However, the situation in Belgium is a bit complex, as several different vaccines with different compositions have been included in the schedule at different times. Also, the booster doses given to adolescents and adults have always contained pertactin. Moreover, there are regional policy differences between Flanders and the Brussels-Capital Region and Wallonia. In this study, most of the strains included were from Flanders; this could also be reflected in our findings. No significant difference in pertactin expression or genotype distribution was seen between the different regions. Another explanation could be due to population immunity. During the COVID-19 pandemic, circulation of pertussis was much lower than the pre-pandemic period, resulting in low exposure to the bacterium and low natural boosting of immunity in the population. Post-pandemic, this lowered immunity may have allowed some strains to spread quickly among the adult population, with less selective advantage for pertactin deficiency.

It remains to be seen whether and when Prn-deficient strains will reappear in this country, although a comeback seems likely in light of the partial return to pertactin-containing vaccines (and their continued use in several other European countries).

Figure 3 offers a graphical representation of the different pertactin genotypes found in the Belgian strains over the years. Several different mechanisms causing a lack of pertactin expression can be seen. Insertions of IS*481* within the *prn* gene occur most frequently (37% of cases). Such insertions have been described as an important cause of pertactin deficiency on multiple occasions (2, 12, 22, 23). In fact, for the period 1998–2015, it was shown to be the most frequent cause of it in European strains (2).

Another mechanism, occurring in 25% of the pertactin-deficient strains, is the large deletion of the first 1,340 bp of the *prn* gene and part of the promoter region (1,632 bp total). This same deletion has been described previously in Spain and the USA (23, 24). Moreover, one strain (BORD1824, BIGSdb ID 5165) was found with a similar but smaller 1,083 bp deletion (part of the promoter and the first 800 bp of *prn*).

Point mutations leading to a premature stop codon also caused pertactin deficiency in 16 strains (9%). All three mutations have been described previously (3, 22, 25).

In 28% of the pertactin-deficient strains, an intact *prn2* allele was found. About 70% (*n* = 36) of these showed a known inversion of part of the *prn* promoter region (16). In three strains, a large part of the *prn* promoter was deleted. Finally, 12 strains (7% of all tested pertactin-deficient strains) had an intact *prn2* allele as well as an intact promoter region, the cause of the pertactin deficiency remaining unknown so far. It is possible that other regulatory regions are involved.

Phylogenetic analysis shows that these different mutations are mostly spread out among the circulating strains, without any particular mutation limiting itself to a specific clade. A larger study of the mutations found in Prn-deficient strains from other countries and their phylogenetic relationships could shed more light on pre- and post-COVID transmission patterns.

An important limitation of this study is the limited sample size of typed strains (*n* = 416) out of almost 3,000 PCR-positive cases over the 10-year study period. A significant selection bias is introduced as a result of the requirement of successful culture for further typing, which is typically only achieved for strong positive samples. Future developments allowing for WGS directly on clinical samples may alleviate some of this bias.

In conclusion, our findings illustrate the rapidly changing and sometimes unpredictable nature of pertussis epidemiology, highlighting the need for continued surveillance. Further research should be done to identify the factors that influence pertactin expression in circulating *B. pertussis* strains, as well as assess potential effects on clinical presentation and severity.

## Data Availability

WGS read data have been deposited to ENA under project accession PRJEB88325. Genome assemblies are all available on the BIGSdb Bordetella cgMLST database.
